# Coumarin Derivatives Solvent-Free Synthesis under Microwave Irradiation over Heterogeneous Solid Catalysts

**DOI:** 10.3390/molecules22122072

**Published:** 2017-11-28

**Authors:** Souad Bouasla, Juan Amaro-Gahete, Dolores Esquivel, M. Isabel López, César Jiménez-Sanchidrián, Mabrouk Teguiche, Francisco J. Romero-Salguero

**Affiliations:** 1 Skikda Higher School of Technological Education, Azzaba 21300, Algeria; 2 Departamento de Química Orgánica, Instituto Universitario de Investigación en Química Fina y Nanoquímica IUIQFN, Facultad de Ciencias, Universidad de Córdoba, Campus de Rabanales, Edificio Marie Curie, E-14071 Córdoba, Spain; q22amgaj@uco.es (J.A.-G.); marisa_lopez_martinez@hotmail.com (M.I.L.); qo1jisac@uco.es (C.J.-S.); 3 Applied Chemistry Laboratory (ACL), Faculty of Mathematics, Informatics and Materials Sciences, Department of Materials Science, Guelma 24000, Algeria; teghichemab@yahoo.fr

**Keywords:** phenol, ethyl acetoacetate, solvent-free, microwave irradiation, heterogeneous acid catalysts

## Abstract

A suitable methodology of synthesis of coumarin derivatives by Pechmann reaction over heterogeneous solid acid catalysts in a free solvent media under microwave irradiation is described. Resorcinol, phenol and ethyl acetoacetate were selected as model reactants in the Pechmann condensation. The catalytic activity of several materials—Amberlyst-15, zeolite β and sulfonic acid functionalized hybrid silica—in solvent-free microwave-assisted synthesis of the corresponding coumarin derivatives has been investigated in detail. 7-Hydroxy-4-methylcoumarin and 4-methylcoumarin were obtained in 97% and 43% yields, respectively, over Amberlyst-15. This was the most active catalyst in the Pechmann reaction under studied conditions.

## 1. Introduction

Coumarins are an important family of oxygen-containing heterocycles with a 1-benzopyran-2-one moiety, originally found as secondary metabolites in some plants (*Rutaceae* and *Umbelliferae* family) and microorganisms [1]. Coumarin derivatives have displayed widespread therapeutic applications including antibacterial [2,3], anti-inflammatory [4], antioxidant [5], anticoagulant [6], anticancer [7,8] and anti-HIV [9], among others [10,11]. Additionally, these compounds have attracted a great interest in other fields, such as fragrance and cosmetic [12], agrochemical [13], food and dyes industries [14].

Due to their applicability, several methods have been developed over the years to synthesize coumarin derivatives such as Pechmann condensation [15], Knoevenagel [16], Perkin, Witting, Reformantsky reactions and by flash vacuum pyrolysis [17] or via carbon suboxide [18]. Among these methods, the Pechmann reaction is a simple and widely used method for the synthesis of 4-substituted coumarins, since it requires simple and inexpensive starting materials, i.e., phenols and β-ketoesters. This reaction proceeds in acidic media, using either homogenous Brönsted acid catalysts like H_2_SO_4_, HCl, H_3_PO_4_ and CF_3_COOH [19] or Lewis acid catalysts such as InCl_3_, Sm (NO_3_)_3_, ZrCl_4_, BiCl_3_ and FeF_3_, among others [20,21,22,23]. However, these catalysts require drastic conditions, long reaction times and large quantities to reach high yields. In addition, they are corrosive, hard to remove from the reaction mixture, non recoverable and pollutants for the environment.

Recently, a number of heterogeneous solid acid catalysts like, Nafion-H, zeolite H-BEA [24], montmorillonite clays [25], ionic liquids [26], Nafion resin/silica nano-composites [27], silica supported perchloric acid (HClO_4_·SiO_2_) [28], mesoporous zirconium phosphate (m-ZrP) [28], cellulose sulfuric acid [29], ZrOCl_2_·8H_2_O/SiO_2_ [30], and many more have been employed to replace classical acids in Pechmann condensation [31,32,33]. The use of heterogeneous acid catalysts presumes advantages like their easy recovery by simple filtration, reusability, less corrosion issues, minimized waste contamination and environmental-friendliness.

On the other hand, in recent years, the concept of speeding up the synthetic transformations by microwave activation has created a lot of interest in organic synthesis [34]. The coupling of microwave heating under solvent-free conditions in chemical processeses catalyzed by solids gives rise to an enhancement of the reaction rate, ease of work-up and high yields [35].

The aim of this work is the optimization of a suitable methodology to yield coumarin derivatives under solvent-free conditions using microwave irradiation. The catalytic activity of several heterogeneous solid acid catalysts in the Pechmann reaction of phenols (resorcinol and phenol) with ethyl acetoacetate to produce 7-hydroxy-4-methyl coumarin and 4-methylcoumarin, respectively, has been studied in detail.

## 2. Results

**Scheme 1:**
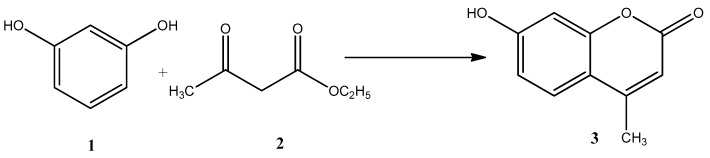
Pechmann reaction of resorcinol (**1**) with ethyl acetoacetate (**2**) to produce 7-hydroxy-4-methylcoumarin (**3**).

Firstly, resorcinol and ethyl acetoacetate were employed as the model reactants in the Pechmann condensation under microwave reaction. Resorcinol (1,3-dihydroxybenzene) was chosen due to its high reactivity (Scheme 1). 

Commercial sulfonic acid resin, Amberlyst-15, was employed to evaluate the reaction kinetic with the above-mentioned reactants (1:1 molar ratio) at 100 °C. After 5 min of microwave irradiation, Amberlyst-15 showed a conversion of around 83%. By varying the reaction time from 5 min to 20 min, a maximum yield of 97% was obtained (Table 1). No reaction was observed in the absence of catalyst (blank reaction).

The catalytic behavior of Amberlyst-15 in the synthesis of 7-hydroxy-4-methylcoumarin was compared with other acid solid catalysts, such as zeolite H-β and sulfonic acid functionalized hybrid material (TS-OS-SO_3_H). The results are summarized in Table 1. Amberlyst-15 catalyst exhibited much higher yield of 7-hydroxy-4-methylcoumarin than the other catalysts after 20 min of reaction. Compared with the Amberlyst-15, the H-β and TS-OS-SO_3_H materials contained fewer acid sites per gram of catalyst (Table 1). In general, the catalytic activity on the synthesis of 7-hydroxy-4-methylcoumarin could be related to the amount of acid sites [36]. However, the sulfonic acid functionalized hybrid silica with an acidity of 1.24 mmol H g^−1^, four times lower than Amberlyst-15, exhibited a yield higher than expected. This suggests that not all sites in the interior of the resin are accessible to the reactants. Additionally, this phenomenon could explain the double yield (44%) obtained over TS-OS-SO_3_H in comparison with the corresponding β zeolite (21%). Despite a similar value of BET surface area and acidity (Table 1), probably the diffusion of resorcinol, a meta-hydroxy substituted phenol, is hindered through the zeolitic channels [27]. Another zeolite with higher Si/Al ratio (zeolite H-β, Si/Al = 75) was evaluated in this reaction. This showed similar conversion to H-β, which indicated that hydrophobicity does not have any important role in this type of reaction [37].

It is well known that, of the simple mono-, di-, and tri-hydric phenols, resorcinol is the most reactive, and it easily condenses with many substituted and cyclic β-ketonic esters. Unlike resorcinol, phenol exhibits low reactivity giving only ca. 3% yield of 4-methylcoumarin on the Pechmann reaction with ethyl acetoacetate in the presence of sulfuric acid [38]. Earlier reports on microwave-assisted solvent-free synthesis of 4-methylcoumarin using nano-crystalline sulfated-zirconia catalyst did not show product formation under the studied conditions [39]. Due to the scarce studies of the synthesis of 4-methylcoumarin, phenol was selected as the reactant model to optimize the experimental catalytic conditions under microwave irradiation.

### 2.1. Optimization of Reaction Conditions

**Scheme 2:**
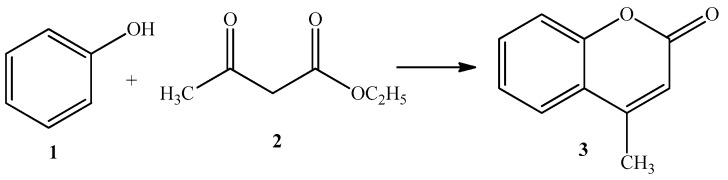
Synthesis of 4-methylcoumarin (**3**) via the Pechmann condensation of phenol (**1**) and ethyl acetoacetate (**2**).

The Pechmann condensation of phenol and ethyl acetoacetate to afford 4-methylcoumarin on microwave-assisted solvent-free conditions was studied using different acid heterogeneous catalysts (Scheme 2). Initially, the experimental conditions such as the amount of catalyst and temperature reaction were optimized over Amberlyst-15.

**Figure 1:**
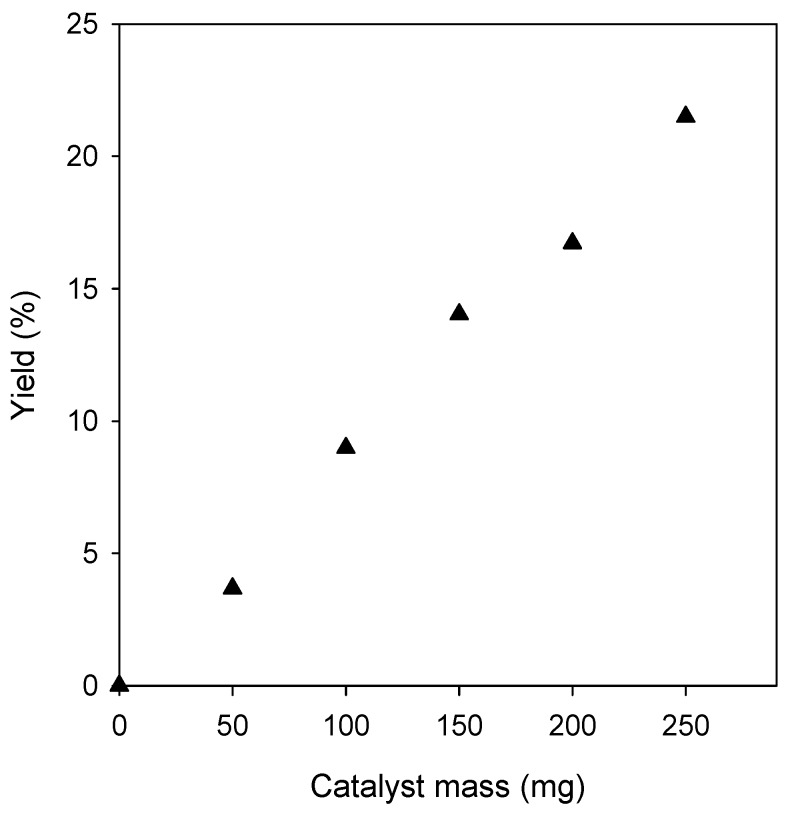
Effect of catalyst loading on the yield of 4-methylcoumarin: reaction time, 20 min; reaction temperature, 100 °C; and phenol/ethyl acetoacetate, molar ratio 1:1.

To optimize the amount of catalyst, the Pechmann condensation between phenol and ethyl acetoacetate was carried out under solvent-free conditions over Amberlyst-15 catalyst for 20 min at 100 °C using a molar ratio of phenol: Ethyl acetoacetate of 1:1. As can be observed, the conversion of phenol gradually increased from 3.8% to 21.5% with increasing the amount of catalyst from 0.050 to 0.250 g (Figure 1). Subsequently, the effect of the temperature in the reaction was also studied. When the reaction was conducted at 130 °C, a total conversion of phenol around 43% was obtained, which is twice the conversion reached at 100 °C (see Figure 1, 21.5%). A further increase of the temperature at 160 °C gave a conversion of 54%. However, it was accompanied with a lower selectivity, giving rise to the formation of a high number of byproducts. Accordingly, for further experiments, the reaction temperature was set up at 130 °C.

At the same microwave conditions (130 °C, 0.250 g Amberlyst-15, 20 min), the Pechmann reaction was performed under conventional thermal heating. After 20 min, the phenol conversion was not higher than 18%. This result confirms the potential of microwave irradiation in this type of reaction as an alternative to conventional heating methods.

The catalytic performance of Amberlyst-15 was compared with those of H-β and TS-OS-SO_3_H (Table 2). After a contact time of 20 min, 4-methylcoumarin was obtained in yields of 13% and 10%, respectively. Again, the highest conversion was obtained over the polysulfonated resine due to its highest amount of acid centers. In this case, the 4-methylcoumarin yield over H-β was similar to the obtained over TS-OS-SO_3_H material, which indicated the better diffusion of the phenol, a mono-hydric phenol, through the pores of the zeolite.

### 2.2. Reaction Mechanism

**Scheme 3:**
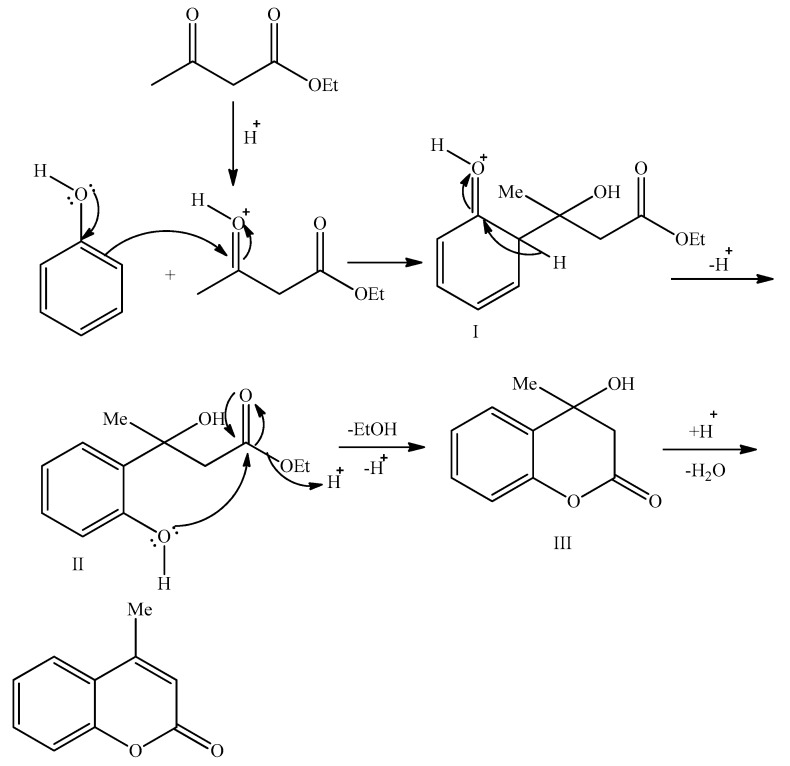
A plausible mechanism for the Pechmann condensation of phenol and ethyl acetoacetate over acid catalysts.

The plausible reaction mechanism for the Pechmann reaction of phenol with ethyl acetoacetate was elucidated based on HPLC-MS analysis. The presence of 4-methylcoumarin and its intermediates in the reaction mixture was confirmed by HPLC-MS measurements. The reaction pathway follows the mechanism proposed by Robertson et al. [40] as illustrated in Scheme 3. Ethyl acetoacetate is chemisorbed on the acid sites of the catalyst. The cation, thus formed, is a good electrophile and reacts with phenol to generate a first intermediate I. The subsequent readsorption of the intermediate on the acid site of the catalyst yields the second intermediate II with *m*/*z* = 225.10. Then, an intra-molecular transesterification reaction results in ring closing, giving rise to the third intermediate III with *m*/*z* = 149. Finally, its dehydration produces the final product, 4-methylcoumarin.

## 3. Materials and Methods 

### 3.1. Chemicals

Ethyl acetoacetate (≥99%), resorcinol (≥99%), phenol (≥99%), dodecane (≥99.8%) and Amberlyst-15 were purchased from Sigma-Aldrich (St. Louis, MO, USA). Absolute ethanol was provided by Panreac (Barcelona, Spain). The NH4-β (Si/Al = 12.5 and Si/Al = 75) was purchased from Zeolyst International company (Kansas, KS, USA). It was calcined at 600 °C (at a rate of 1 °C/min) for 3 h to obtain the protonic form (H-β). TEOS (tetraethyl orthosilicate), BTEPTS (bis[3-(triethoxysilyl)propyl]-tetrasulfide) and Brij-76 (i.e., polyoxyethylene(10)stearyl alcohol) were purchased from Aldrich.

### 3.2. Synthesis of Sulfonic Acid Functionalized Hybrid Silica (TS-OS-SO_3_H)

Sulfonic acid functionalized hybrid silica (TS-OS-SO3H) was synthesized according to our previous method by using TEOS and BTEPTS as the silica and organosilica precursors [41]. A molar ratio of the precursors of 75:25 was added to the synthesis mixture containing Brij 76 (6 g), HCl (19.6 mL) and H_2_O (279 mL). The solution was stirred for 24 h at 50 °C, and, then, the suspension was aged at 90 °C for 24 h under static conditions. The resulting solid was recovered by filtration and dried in the air. The surfactant was removed by successive extraction processes by heating the material at reflux in a HCl solution (1 mL of 37% HCl and 50 mL of ethanol, per gram of solid) for 12 h. To oxidize the tetrasulfide moieties to sulfonic acid groups, 0.3 g of material was stirred with 10 mL H_2_O_2_ (30%) at 50 °C for 3 h. Later, the solid was washed with 25 mL of 1.2 M H_2_SO_4_ and, then, washed with water until a neutral pH was obtained. Finally, the solid was dried at 100 °C under vacuum.

### 3.3. Microwave Assisted Solvent-Free Synthesis of Coumarin Derivatives

Solvent-free synthesis of coumarin derivaties was carried out by using microwave irradiation (1000 W) in a FlexiWave Milestone Lab Microwave (Milestone Srl, Sorisol (BG), Italy) A 50 mL microwave reaction tube equipped with a magnetic stirring bar (stirring capacity was set to 80%) was charged with a mixture of resorcinol or phenol (10 mmol), ethyl acetoacetate (10 mmol) and 0.250 g of heterogeneous catalyst. The reaction mixture was kept in a microwave sealed tube at 130 °C for 20 min. The progress of the reaction was monitored by thin layer chromatography (TLC). The resulting mixture was cooled to room temperature, treated with absolute ethanol and then filtered to recover the catalyst. The reaction sample was analyzed by GC-MS using dodecane as an internal standard. After evaporation of the solvent in a rotary evaporator, the crude solid was recrystallized in aqueous ethanol to give the desired coumarin. Melting points were recorded on Stuart Electrothermal Capillary melting point apparatus and are uncorrected. Pure products: 7-Hydroxy-4-methyl coumarin (white solid, m.p. = 184.5 °C), 4-methyl coumarin (yellow solid; m.p. = 84 °C).

## 4. Conclusions

In summary, we have optimized the synthesis of 7-hydroxy-4-methylcoumarin and 4-methylcoumarin by Pechman reaction of resorcinol and phenol with ethyl acetoacetate by using heterogenous acid solid catalysts under solvent-free microwave irradiation. The catalytic performance of Amberlyst-15, zeolite-β and sulfonic acid functionalized hybrid silica, with different acidity and surface properties, was evaluated. Among them, Amberlyst-15 showed much higher activity due to its high density of acid centers.

## Figures and Tables

**Table 1:** Condensation reaction of resorcinol with ethyl acetoacetate using various heterogeneous solid acids catalysts ^1^.

Catalyst	S_BET_^2^ (m^2^g^−1^)	Acidity (mmol g^−1^)	Yield (%)
Amberlyst-15	43	4.30	97
H-β	530	1.01	21
TS-OS-SO_3_H	448	1.24	44

^1^ Reaction conditions: Molar ratio (resorcinol/ethyl acetoacetate) = 1:1, catalyst amount = 0.050 g, temperature = 100 °C, reaction time = 20 min. ^2^ Specific surface area determined using the BET (Brunauer-Emmett-Teller) method over a relative pressure (P/P_0_) range of 0.05–0.30.

**Table 2:** Condensation reaction of phenol with ethyl acetoacetate with different heterogeneous catalysts. ^1^

Catalyst	Yield (%)
Amberlyst-15	43
H-β	13
TS-OS-SO_3_H	10

^1^ Reaction conditions: Molar ratio (phenol/ethyl acetoacetate) = 1:1, catalyst amount = 0.250 g, temperature = 130 °C, reaction time = 20 min.
